# Academy of Stomatology

**Published:** 1895-12

**Authors:** George D. B. Darby

**Affiliations:** Secretary


### ACADEMY OF STOMATOLOGY.

*Tuesday, October 15, 1895.*

THE President, Dr. Guilford, called the meeting to order, and stated that this was a special meeting, called for the purpose of hearing a paper read which was written by Dr. Fillebrown, of Boston, and one also from Dr. Custer, of Ohio.

The president stated that Dr. Fillebrown was unable to be present, and also that some of the things which were to be used to illustrate the points in the paper had not arrived.

The paper was read by the secretary.

(For Dr. Fillebrown's paper, see page 731.)

DISCUSSION.

Dr. Darby, in explanation of Dr. Fillebrown's absence and of the apparatus which it was expected would be here, said,—

Just before I came to the meeting Dr. Kirk called at my house and stated that the apparatus had not reached here. It was forwarded by express, but up to the time of his leaving the dental depot, where it was to have been sent, it had not arrived, but I understood him to say there would be a large drawing of the apparatus here that you could see.

He also wished me to ask for some expression of opinion from the members of this society on this subject, believing that it was applicable to dental practice. Those of you who have been in the habit of administering either chloroform or ether—especially ether—to patients, and have taken advantage of the first stages of etherization in excavating sensitive teeth, know that it is of great value to the dentist in dealing with young and nervous persons.

Years ago, when I had more children to operate for than now, my son having taken most of that kind of practice, I frequently felt the necessity of etherizing my patients. Very frequently I would administer enough to give them courage and to make them less obstreperous. I found the greatest benefit to myself and freedom of pain to my patients by putting them under the first stages of etherization.

Now, if one can carry this along with this apparatus of Dr. Fillebrown's,—and I cannot see any reason why they are unable to throw a spray or vapor of ether into the patient's face eight, ten, or fifteen inches from your own, so that you will not anæsthetize yourself,—it seems to me it would be a great help in dealing with patients, either men or women. I know of a physician who will not submit to having a tooth filled that is at all sensitive until he etherizes himself, and he always demands a bottle of ether and an inhaler that he may take some when about to have a sensitive tooth excavated. That seems almost childish, and yet there are people who dread pain so much that they would rather etherize themselves, and even submit to the nausea which sometimes follows the full anæsthetic effect, than suffer the pain.

If we could keep our patients about half drunk on ether while excavating the teeth by this method, and by not anæsthetizing to absolute and dense drunkenness, I don't know but it would be a good thing to put into our practice.

I hope, for the benefit of Dr. Fillebrown and others, we may hear some remarks on the paper.

*Dr. Thomas.*—I agree with Dr. Fillebrown that for operations, such as excavating sensitive teeth, it would be an excellent thing. There is one objection to putting it in use in practice. Dr. Fillebrown acknowledges that it is impossible, or next to impossible, to get a patient thoroughly under the influence of ether with the spray or with the current, so that it is necessary to produce anæsthesia, first, by the usual method of placing a face-piece over the patient, and then keeping up the anæsthesia after it has been established. In that way I think it would be detrimental to one's practice in filling teeth and working around the mouth.

I can very readily see or understand that, when the condition of anæsthesia is produced to the point of unconsciousness, it could be very readily kept up, and it is an excellent method, in my mind, for doing so. It is not the ether vapor we want as much as it is the etherized air, and with an apparatus such as this, I do not see why anæsthesia could not be maintained almost indefinitely for operations in the mouth.

I hardly see where it is of any advantage to a dentist in the extraction of teeth, for the reason that it takes too long. If I had to keep a patient under it for twenty minutes to extract a tooth, I should want to follow another occupation. I have on frequent occasions, in giving nitrous oxide, first brought the patient to a state of anæsthesia, and then it has been kept up indefinitely by the administration of ether. It is a quicker and pleasanter way, by far, for operations extending over a length of time. By producing anæsthesia by nitrous oxide, it could be followed up by the etherized air which Dr. Fillebrown has introduced.

The danger of giving anæsthetics is more from asphyxiation than from the effects of the anæsthetic. Anæsthesia, to my mind, is nothing more nor less than intoxication, whether by chloroform or ether, or anything else. The physiological condition is the same on the patient as is that effected by alcohol, one produced by inhalation and the other by alimentation. I don't see any more danger in giving ether than in a person getting drunk by liquor; the danger, except in idiosyncrasies, is from the want of oxidation. There are many cases that cannot bear this; they show symptoms of depression of the heart and symptoms of asphyxiation very rapidly. You have seen cases, probably, where a teaspoonful of brandy would intoxicate; others it would not so affect. There are those that anæsthesia would not affect as quickly. Take chloroform: I have known instances where even placing a piece of cotton saturated with chloroform in the tooth of the patient produced unconsciousness. The specific effect caused by a particular anæsthetic, no matter what it is, to the same person, would be just as readily affected by the use of alcohol, so that sometimes dangers are produced more by asphyxiation than from the effects of the anæsthetic, whether vapor of ether, chloroform, or nitrous oxide. The element of danger is in the want of oxidation.

In Dr. Fillebrown's current the air is etherized, as he expresses it. I think, possibly, it would be better for him to have the current of air go through the ether rather than over it.

A man may be drunk on alcohol enough to be comfortable, or he may be drunk enough to paralyze himself. There is a difference between intoxication and paralysis. I remember on one occasion of giving gas to a patient in a case of laparotomy, and the young man, a physician, who was giving the ether, was much more interested in what was being done by the surgeon than in administering the ether, and I believe if I had not been there the patient would have died. The tongue had fallen back in the mouth and it came very near resulting fatally. If he had been watching the patient, as I do in giving nitrous oxide, and had taken away the plate or opened it to admit a little air as soon as there was any indication of asphyxiation, he could have carried the patient on indefinitely.

I should think the apparatus would be useful in long operations.

Dr. Cryer was called upon, but declined to enter into the discussion, as he had not heard the paper read.

*Dr. Burchard.*—I don't know anything, practically, about anæsthetics. I have seen, as Dr. Darby mentioned, in the early stage of intoxication, teeth extracted and minor surgery performed absolutely without pain and without any reaction at all.

But there is one point Dr. Thomas spoke of, and that is of anæsthesia being a purely functional disorder. It is more than that.

[The doctor then gave an explanation of the effects produced by etherization, the evidences of albuminuria, etc., stating that he considered the method of Dr. Fillebrown one of great usefulness as far as the primary stages of etherization are concerned.]

Dr. Porter asked of Dr. Thomas the question, How is it that, in administering nitrous oxide, we are more successful when we give the nitrous oxide without any admission of air?—that is, during its administration, not subsequently. Very often when any air gets into the system we are unsuccessful, when with a continued current of nitrous oxide we have almost perfect anæsthesia and are successful.

Dr. Thomas answered, In many cases asphyxiation can be obliterated entirely by the admission of a certain amount of atmospheric air, the inhalation being not continuous, but alternate. I sometimes let go the nose or raise the lip to let a little air enter, and thus anæsthesia will be produced of a more profound character, and the patient will be under better control by the admission of air during inhalation than without it. If you exclude air entirely, you carry the patient to absolute unconsciousness. The convulsive action, the twitching and jerking, is entirely obviated by the admission of air to a certain extent. You can admit it according to the temperament of the patient, and it will relieve all that jactitation, jerking, twitching, and convulsion, and the patient will be carried into an absolute and profound state of anæsthesia much more satisfactorily than with nitrous oxide absolutely pure.

Dr. Porter then continued as follows: In regard to this question I think we have many perplexing problems. We all know the vivid dreams in the inhalation of nitrous oxide; we know the convulsion, we know the jactitation, and that is hyperæsthesia in one sense of the term; it is energy; it is motion; it is force. Here we have a force, a product of something poisonous, when force is generally a product of life, the product of activity.

When we look at the question in the light of modern science we find the difficulty vanishes altogether. The hyperæsthesia of nitrous oxide or ether inhalation is due to a current of energy circulating through the system and centring in one part. Now, that current is at the expense of the energy of the other parts. It is at the expense of the energy of the nerves of the tooth; it does not respond to any stimulus of any kind. There is no pain because there is no response, and it just lies in that word,—response.

We find it illustrated in many ways in physics: when we touch a violin string, it will respond to a touch, to the slightest stimulus; it stimulates the air and vibrates hundreds of yards away. We find it illustrated in many mechanical ways. Now, how is that response effected? It is effected because there is nothing in the earth or nature dead; we have activity in everything. In what we call dense matter or dead tissue, it is only dead apparently; there are energies, and these energies are opposing each other. That is the whole secret of the matter. In giving air with gas I have frequently had difficulty in extracting teeth, while the patient suffered considerable pain; in one or two cases I have neglected to hold the nose and have had considerable difficulty. The reason is simply this: in the continued response of the cells of the brain there is that combination of energies together; there is that response.

At this point Dr. Burchard stated that he did not think the remarks made by Dr. Porter were entirely pertinent, and he thought Dr. Porter was confusing terms.

After some conversational discussion between Dr. Porter and Dr. Burchard, the president stated that while the matter could be very profitably discussed at another time, it was not in the line of the paper read, and it would be well to confine the discussion to the paper.

Dr. Pearsol, of Dublin, was next called upon by the president, and made the following remarks:

It does me great honor to be called upon to say a few words on this matter, because anæsthetics have exercised our minds over on the other side of the water as they have yours on this side.

There are two schools of anæsthesia at home. One contends that chloroform is the only road to salvation, and the other that ether is the direct course; but there is a mean between the two. It is not uncommon to find that a person with a large experience in the use of chloroform is involved in difficulty in administering ether, and one familiar with the use of ether fails to administer chloroform. A man who gives ether properly ought to have the patient ready in about three minutes; sometimes I have seen it done in a minute and a half; it may go on for five minutes, but I think the time mentioned by Dr. Fillebrown is all wrong.

We use it in the dental hospital in Dublin a great deal for the extraction of teeth; more than nitrous oxide, and the reason is simply that it is so much cheaper to anæsthetize with ether than with nitrous oxide; nitrous oxide costs about two shillings a head, whereas ether is fourpence, and as we are all poor people, we have to take the ether. In operating we issue directions to patients, so that we do not have the same trouble as formerly. A patient purchases an anæsthetic card beforehand, and makes an appointment for a certain time in the morning, and they are recommended to come fasting, because it is a well-known fact that this is a great advantage in administering anæsthetics. I have seen the most distressing scenes with patients who, if they had had the stomach empty, would have had no trouble at all.

In Dublin we are very fortunate in many ways, because we have some excellent administrators of anæsthetics among the medical and surgical professions, and we have very few deaths in the year. We have not had more than one death in five or six years past, while in London, on the contrary, where chloroform is much more used than ether, they are having an epidemic of deaths. If you read the *Lancet*, you will observe how many deaths have occurred on the operating-table. I cannot tell the reason.

Dr. Hewitt, of London, has certainly made a great advance with the method which he uses. In the cases in which I saw him operate there was no crying out nor shedding of tears, such as we often see. I think Dr. Hewitt has recently made his apparatus very portable; simply a bottle of oxygen and a bottle of nitrous oxide, with a little foot-piece with which you can turn it off and on, and a chamber for mixing it in. It used to be very cumbrous, but he now has it in convenient form.

I can say no more except that I am glad to see you are travelling in the same direction as we do at home. My experience teaches me that if a man will give ether with the chloroform method, he will have trouble; but if we have a chloroform man to give chloroform to the young and an ether man to give ether to adults, we can save a great deal of trouble and time.

I do not usually attempt anæsthetic work in my office, but generally in a patient's room, because then if the patient is sick at all it is in his house and not in my room, and I think it is under more favorable conditions.

Dr. Custer followed with his paper on electrical heating, accompanied by experiments. These were interesting and effective, but were not of a character to elicit discussion. A few questions were asked by the members, which were answered by the essayist.

Dr. McQuillen stated that the members of the society were invited to attend the November meeting of the Odontological Society of New York City, and that Dr. George D. B. Darby would read a paper at that time.

*Dr. Truman.*—Before we adjourn I desire to express my appreciation of the exhibit of Dr. Custer we have seen here to-night, in which I have been very much interested, and feel it due to the exhibitor to express my thanks.

As I sat here and recalled my early boyhood days, when I used to sit in the cellar by the furnace for the purpose of manufacturing porcelain teeth, with all the difficulties of heating, and then to be translated from that time to this table with the same process going on within a flask of a few inches in diameter, it seems to me all an indication of the great progress that has been made, not only in dentistry but in everything, in science and art. Therefore I feel it a gratification to express my appreciation, and I move that a vote of thanks be tendered Dr. Custer, by this society, for the careful work of this evening.

Dr. Peirsol also expressed the great satisfaction with which he had witnessed the experiments, and hoped there would be some way to have these exhibited in England before the dental societies.

Dr. Burchard seconded the motion of Dr. Truman and spoke of the great advance that had been made as illustrated by the experiments of the evening.

On motion, adjourned.

George D. B. Darby,
*Secretary.*

